# Morphine effects on striatal transcriptome in mice

**DOI:** 10.1186/gb-2007-8-6-r128

**Published:** 2007-06-28

**Authors:** Michal Korostynski, Marcin Piechota, Dorota Kaminska, Wojciech Solecki, Ryszard Przewlocki

**Affiliations:** 1 Department of Molecular Neuropharmacology, Institute of Pharmacology PAS, Smetna 12, 31-343, Krakow, Poland

## Abstract

**Background:**

Chronic opiate use produces molecular and cellular adaptations in the nervous system that lead to tolerance, physical dependence, and addiction. Genome-wide comparison of morphine-induced changes in brain transcription of mouse strains with different opioid-related phenotypes provides an opportunity to discover the relationship between gene expression and behavioral response to the drug.

**Results:**

Here, we analyzed the effects of single and repeated morphine administrations in selected inbred mouse strains (129P3/J, DBA/2J, C57BL/6J, and SWR/J). Using microarray-based gene expression profiling in striatum, we found 618 (false discovery rate < 1%) morphine-responsive transcripts. Through ontologic classification, we linked particular sets of genes to biologic functions, including metabolism, transmission of nerve impulse, and cell-cell signaling. We identified numerous novel morphine-regulated genes (for instance, *Olig2 *and *Camk1g*), and a number of transcripts with strain-specific changes in expression (for instance, *Hspa1a *and *Fzd2*). Moreover, transcriptional activation of a pattern of co-expressed genes (for instance, *Tsc22d3 *and *Nfkbia*) was identified as being mediated via the glucocorticoid receptor (GR). Further studies revealed that blockade of the GR altered morphine-induced locomotor activity and development of physical dependence.

**Conclusion:**

Our results indicate that there are differences between strains in the magnitude of transcriptional response to acute morphine treatment and in the degree of tolerance in gene expression observed after chronic morphine treatment. Using whole-genome transcriptional analysis of morphine effects in the striatum, we were able to reveal multiple physiological factors that may influence opioid-related phenotypes and to relate particular gene networks to this complex trait. The results also suggest the possible involvement of GR-regulated genes in mediating behavioral response to morphine.

## Abstract 2

Global transcriptional analysis of mouse striata following acute and chronic exposure to morphine reveals multiple physiological factors which may affect opioid-related phenotypes and implicates a number of gene networks, including glucocorticoid receptor regulated genes, in the response to this opioid.

## Background

Opioids are considered to be among the most potent drugs for relieving severe chronic pain. Long-term morphine treatment is undesirable because of the development of tolerance to its analgesic effects and physical dependence. On the other hand, prolonged abuse of opiates leads to drug addiction - a chronic, relapsing disorder with a complex mechanism. Accumulating evidence is converging to suggest that formation of opioid addiction involves changes in synaptic structure and neuronal plasticity [1]. These long-lasting neuroadaptations probably include compound changes in gene expression in the mesocorticolimbic system of the brain [2]. The major substrates of the molecular and cellular mechanisms of opioid addiction are suggested to be the dorsal and ventral striatum. Morphine administration enhances the release of dopamine in both the dorsal striatum and nucleus accumbens [3]. It is well established that the nucleus accumbens, a ventral subregion that receives dopaminergic projections from the ventral tegmental area, is related to the reward properties of opioids [4]. The dorsal part of the striatum is a brain region that is implicated in habit learning, which is a fundamental component of addiction [5].

It is well known that drugs of abuse stimulate the transcription of numerous genes in several brain regions [6,7]. Moreover, a significant contribution of genetic factors to vulnerability to the addictive action of opiates and other addictive drugs is well established [8]. Several other effects of opioid action, for example analgesia and hypothermia, are also likely to be determined by combinations of genetic factors [9]. In contrast, the influence of genotype on genomic response to opioids and the association between changes in gene expression and development of the rewarding and addictive effects are poorly characterized. Inbred strains of mice with well described phenotypes provide valuable models in which to analyze interactions between genetic background, and behavioral and transcriptional responses to the drug.

To separate the relationship between the different effects of morphine and the gene expression profiles in the striatum, we compared responses to acute and chronic drug treatment across four mouse strains with extreme opioid-related phenotypes (C57BL/6J, DBA/2J, 129P3/J, and SWR/J). Two commonly used inbred strains of mice, C57BL/6J and DBA/2J, exhibit remarkable differences in morphine-induced locomotor activation and conditional place preference [10,11]. Compared with the other strains, C57BL/6J mice were found to have the greatest preference for oral self-administered morphine [12]. Furthermore, the 129P3/J strain failed to develop physical dependence and tolerance, whereas extraordinary sensitivity to opioid withdrawal was observed in SWR/J mice [13].

Our prior comparison of gene expression profiles of naïve animals from the selected inbred mouse strains indicated diversity at the level of several hundreds of transcripts in the striatum [14]. Here, we use microarray technology to obtain a profile of genes that are regulated by acute and chronic morphine in the striatum of the four mouse strains. Our ultimate goal is to link particular genes, regulatory elements, and specific signaling pathways with opioid-related traits. To this end, we have combined gene expression profiling with bioinformatic approaches and behavioral testing. The results presented here identify several novel morphine-responsive genes that may modulate the molecular as well as behavioral response to morphine and may contribute to development of morphine addiction.

## Results

### Microarray analysis

**Figure 1:**
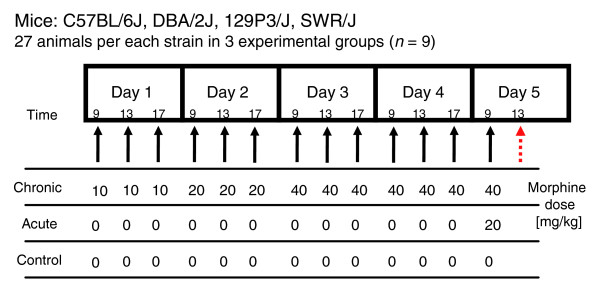
Protocol of morphine administration. Acutely treated mice (*n *= 9) were injected with a single dose of morphine (20 mg/kg, subcutaneously). Chronically treated animals (*n *= 9) were injected with increasing doses of morphine for 5 days (10, 20, and 40 mg/kg, subcutaneously; see Materials and methods). Control (*n *= 9) and acute morphine groups received injections of saline in the same time schedule as the chronic group received morphine. Animals were killed by decapitation 4 hours after the last injection. The time point used for tissue collection and gene expression analysis is indicated by the red arrow.

A microarray experiment was designed to determine the impact of genetic background on the transcriptional effects of morphine in striatum. Twelve experimental groups were compared to analyze the transcriptional response to acute and chronic morphine among the four inbred strains of mice (Figure 1). Three biologic replicates of the microarray were prepared for each experimental group. The quality of microarrays was carefully checked to ensure that hybridization of the samples to the arrays was comparable across the dataset composed of 36 microarrays (see Materials and methods, below). Correlation coefficients of raw microarray results within the strains (129P3/J: 0.986 to 0.988; C57BL/6J: 0.977 to 0.993; DBA/2J: 0.979 to 0.99; and SWR/J: 0.983 to 0.987) and within the experimental groups (control: 0.977 to 0.987; acute: 0.988 to 0.993; and chronic: 0.981 to 0.987) were very high. The microarray data reported in this manuscript are publicly available at the Gene Expression Omnibus database under accession number GSE7762 [15].

Signals from 21,467 probe sets were detected reproducibly on the microarrays, representing 46% of all probe sets on the microarray (23,633 were filtered out). Genes annotated to these probe sets were considered to be expressed in the examined brain tissue. The list of detected probe sets was used for further analyses. Main factors (strain and treatment), as well as interaction, were calculated by using multivariate analysis of variance (MANOVA).

### Strain differences in gene expression profile

The influence of genetic background on gene expression level was estimated. Our previously presented comparison of basal gene expression profiles between C57BL/6J, DBA/2J, 129P3/J, and SWR/J strains identified more than 1,000 transcripts with a different level of mRNA [14]. At present, using similar statistical criteria as previously (false discovery rate [FDR] < 1%, rank > 3), we identified 3,457 probe sets (corresponded to 2,870 different transcripts) with significant inter-strain differences (Additional data file 1). Such a large disparity in the mouse striatal transcriptome was estimated by comparing nine array replicates prepared per strain from all of the treatment groups. More than half of the identified probe sets exhibited markedly significant results (1,735 with rank > 7; see Materials and methods, below, for details). To estimate the quantitative magnitude of the differences between transcriptional profiles, we compared the relative expression level measured for each strain with the mean level of expression from the four strains. Only about 100 probe sets in each strain, from the total of 3,457, were recognized as exhibiting a more than twofold difference over mean value (Table 1).

**Table 1:** Summary of genotype-dependent differences in striatal gene expression profile between mouse strains

Fold change of mean mRNA level^a^	129P3/J strain	C57BL/6J strain	DBA/2J strain	SWR/J strain
>1.2	906 (+295)^b^	925 (+511)	888 (+277)	924 (+391)
>1.5	261 (+50)	230 (+108)	288 (+53)	256 (+60)
>2	99 (+9)	68 (+29)	111 (+14)	89 (+9)
>3	43	20 (+6)	48 (+5)	36

The mouse strains evaluated were 129P3/J, C57BL/6J, DBA/2J, and SWR/J. ^a^Fold change over mean mRNA level from all four strains for 3,457 significantly different probe sets. Relative expression levels were computed from MBEI perfect match/mismatch (PM/MM) data. ^b^Total numbers of probe sets are presented. In addition, numbers of transcripts with increased level comparing to mean are included in parentheses.

However, levels of expression of about 1,000 genes in each strain were found to differ by at least 1.2-fold. About 25% transcripts had higher expression and about 75% had lower expression compared with the other three strains. The observed distribution of differences was similar across the four inbred strains, with the modest exception for C57BL/6J mice. In this strain, similar amounts of transcripts were over and under the mean value.

The contribution of strain or treatment factors to the variation in expression of each gene was described by the portion of multiple regression R^2 ^attributable to strain and morphine treatment as the main effects. Average R^2 ^value was greater for strain effects than for morphine treatment effects. Almost 50-fold more transcripts exhibit R^2 ^above 0.6 for strain effects (2,080) than for treatment effects (43).

### Morphine effects on striatal gene transcription

**Figure 2:**
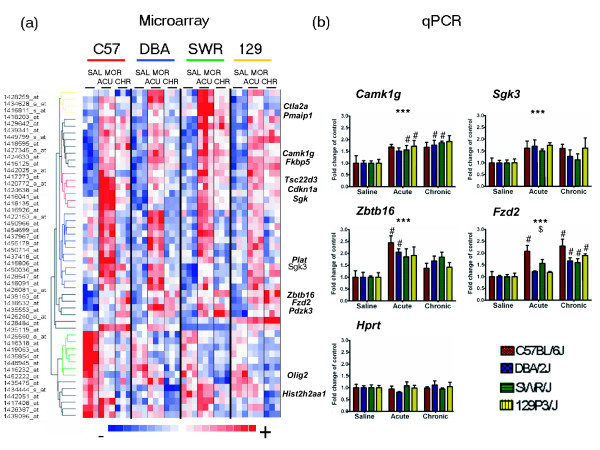
Gene expression changes induced by morphine treatment in the striatum of the four inbred strains of mice. The four strains were C57BL/6J, DBA/2J, SWR/J, and 129P3/J. **(a) **Clustering image of genes whose expression was the most significantly altered, according to microarray analysis (SAL, control group; MOR, acute [ACU] and chronic [CHR] morphine groups). Colored rectangles represent expression levels of the gene indicated by the probe set on the left. Intensity of the color is proportional to the fold change, as indicated on the bar below the cluster image. Hierarchical clustering was performed with the dChip software using Euclidean distance and average linkage method. **(b) **Validation of morphine-induced regulation of expression of the selected genes by quantitative real-time reverse transcription polymerase chain reaction (qPCR). Results are presented as fold change over control group with standard error (*n *= 6 to 9). Significant main effects from multivariate analysis of variance for morphine treatment (***P < 0.001) and interaction (^$^*P *< 0.05) are indicated. Difference between morphine-treated and control groups was analyzed using Bonferroni *post hoc *test (^#^*P *< 0.05).

Morphine treatment altered the expression of 618 transcripts covered by 661 probe sets (FDR < 1%, rank > 3; Additional data file 1). A group of transcripts (56 probe sets) with a high level of significance (rank > 7) was used in hierarchical clustering analysis to present example patterns of genes with altered expression (Figure 2). To validate the results obtained by microarrays, quantitative real-time reverse transcription polymerase chain reaction (qPCR) experiments were performed using aliquots of the nonpooled total RNA. From the list of the most significant 56 probe sets, novel morphine-responsive genes with putative neuronal function were selected. The treatment factor in MANOVA was significant for all of the transcripts examined by qPCR. Equal upregulation (about 1.5-fold to 2-fold) of mRNAs for serum/glucocorticoid regulated kinase 3 (*Sgk3*) and calcium/calmodulin-dependent protein kinase I gamma (*Camk1g*) was observed after acute and chronic morphine exposure in all four strains of mice. Greater than twofold induction of zinc finger and BTB domain containing 16 (*Zbtb16*/*Zfp145*) mRNA after acute treatment was confirmed, and this induction was lower after prolonged administration. Furthermore, an increase in the abundance of the frizzled homolog 2 (*Fzd2*) transcript after chronic morphine exposure was detected in all four strains, with the greatest fold change in C57BL/6J mice (Figure 2).

**Figure 3:**
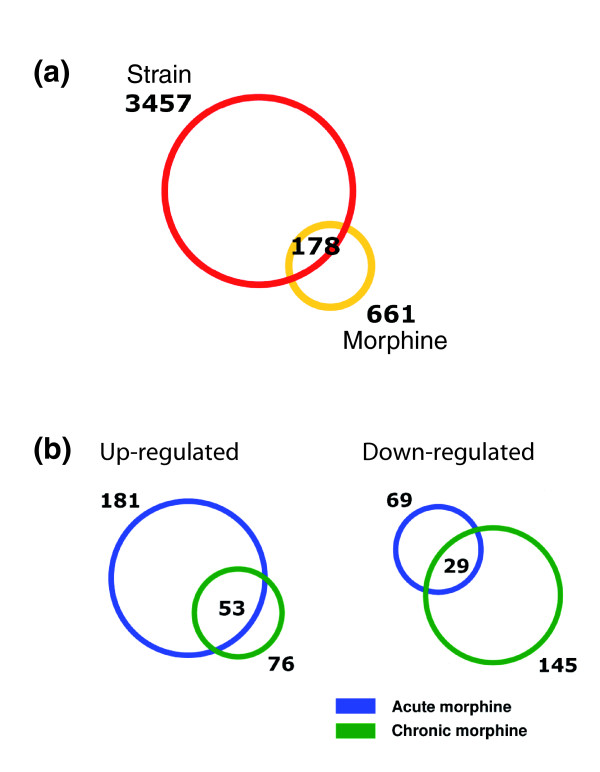
Comparison of the number of genes with expression altered by genotype and morphine treatment. **(a) **In all, 178 probe sets were shared between genes with different levels across the four inbred strains (red circle) and morphine-responsive genes (yellow circle). **(b) **Approximately three-quarters of genes upregulated by chronic treatment (green circles) were also altered by acute morphine treatment (blue circles). On the other hand, 29 genes with a decreased level of mRNA after acute treatment were found in the list of 145 downregulated genes after repeated morphine administration. The list of probe sets with greater than 1.2-fold change over control was analyzed.

Acute and chronic effects of morphine were compared in terms of fold change in the microarray findings (Additional data file 2). The greatest induction of gene expression was observed after acute administration, and this response appeared to be tolerated during further injections of morphine. A total of 181 transcripts exhibited more than 1.2-fold (38 with >1.5-fold) greater expression after acute morphine exposure compared with control groups. In contrast, only 76 (13 with >1.5-fold) were upregulated after chronic treatment (Table 2). Moreover, transcription of about three-quarters of these genes (53) was also activated by acute morphine. Acute morphine reduced the expression of 69 transcripts, and for 29 of them these changes were also observed after repeated injections. Chronic morphine treatment decreased mRNA level of 145 (nine by >1.5-fold) genes in total (Figure 3b).

**Table 2:** Summary of morphine-induced changes in gene expression level in the four inbred strains

Treatment	Fold change over control	All of the four strains	129P3/J strain	C57BL/6J strain	DBA/2J strain	SWR/J strain
Acute upregulation	>1.2^a^	181	179	106	313	255
	>1.5	38	33	39	81	42
Acute downregulation	>1.2	69	44	108	68	67
	>1.5	6	4	25	25	14
Chronic upregulation	>1.2	76	90	116	59	63
	>1.5	13	17	38	8	10
Chronic downregulation	>1.2	145	83	278	98	141
	>1.5	9	9	61	10	12

The mouse strains evaluated were 129P3/J, C57BL/6J, DBA/2J, and SWR/J. ^a^Analysis was performed for the group of transcripts significantly changed after morphine treatment. Relative expression levels for 661 probe sets were computed using MBEI perfect match/mismatch (PM/MM) data.

To identify functional associations between genes with expression that was altered by morphine, a list of probe sets with more than 1.2-fold change over the control group was analyzed by Gene Ontology (GO). Detailed description of the results of GO classification, together with gene names, is included in Additional data file 3. Among the most abundant group of genes (upregulated after acute morphine), functional patterns of transcripts connected with response to abiotic (GO: 0009628; 13 transcripts; *P *= 0.0001) and temperature (GO: 0009266; four transcripts; *P *= 0.004) stimuli were identified as being over-represented. Also, genes associated with negative regulation of cellular processes (GO: 0048532; 17 transcripts; *P *= 0.001) and regulation of apoptosis (GO: 0042981; ten transcripts; *P *= 0.005) were over-represented, including *Sgk3 *and *Zbtb16 *transcripts.

In contrast, acute morphine caused the downregulation of genes involved in nervous system development (GO: 0007399; eight transcripts; *P *= 0.003). At least two transcriptional regulators involved in oligodendrogenesis, namely oligodendrocyte transcription factor 2 (*Olig2*) and GLI-Kruppel family member (*Gli3*), were identified in this group. Downregulation was also observed for genes associated with cell-cell communication (GO: 0007154; 17 transcripts; *P *= 0.008), including the synphilin (*Sncaip*) protein, which is involved in synaptic function.

Repeated opioid administration maintained an increased mRNA level of genes related to negative regulation of apoptosis (GO: 0043066; five transcripts; *P *= 0.002). The decrease in cell-cell signaling genes (GO: 0007267; seven transcripts; *P *= 0.005), including those encoding gap junction membrane proteins α12 and β1 (*Gja12 *and *Gjb1*), was also detected. In addition, chronic treatment decreased the expression of several genes that are involved in nucleosome assembly (GO:0006334; five transcripts; *P *= 0.001). This functional category contains several histone genes, for instance *H1c *and *H2bp*.

### Strain differences in transcriptional response to morphine

Comparison of changes in gene expression profile across the inbred mouse strains with diverse opioid-related phenotypes provides the possibility to find direct associations between transcriptional and behavioral response to the drug. From the list of genes responsive to morphine (661 probe sets), 178 exhibited different levels of mRNA abundance also at basal conditions (Figure 3a). Furthermore, evaluation of the interaction between two main factors (strain and treatment) in MANOVA analysis identified 48 probe sets (rank > 3) for genes with transcriptional difference between the strains in response to morphine. Significant interaction was detected for *Sgk*, *Nfkbia*, and *Hspa1b*, for example (Additional data file 1).

Comparison of the magnitude of inter-strain differences in the level of mRNA across morphine treatments was characterized by fold change (Table 2). Lists of probe sets with more than 1.2-fold and 1.5-fold change in signal for each experimental group were obtained. In general, transcription was mostly increased after a single injection of morphine in all of the strains, whereas repeated administration caused a decrease in the expression levels of a large number of genes. The transcriptional response to acute morphine was the strongest in the DBA/2J strain, whereas chronic morphine affected most the C57BL/6J mice. Furthermore, to characterize the transcriptional representation of biologic processes initiated by morphine in the striatum, ontologic analysis of genes with altered expression was performed (Table 3). Functional annotation was done in each strain on a list of transcripts with a fold change greater than 1.2 (Additional data file 3).

**Table 3:** Examples of significantly enriched GO annotation for the list of morphine-responsive genes

Mouse strain	Morphine treatment	mRNA change	Enriched GO annotation (GO ID)	Number of genes	*P*	Example genes
C57BL/6J	Acute	+^a^	Regulation of apoptosis (0042981)	11	9.1 × e^-06b^	*Tsc22d3*, *Pmaip1*, and *Plekhf1*
129P3/J	Chronic	-	Nucleosome assembly (0006334)	5	1.7 × e^-04^	*Hist1h1c*, *Hist1h3b*, and *Hist1h2bl*
C57BL/6J	Chronic	-	Transmission of nerve impulse (0019226)	10	8.6 × e^-04^	*Sncaip*, *Adora2a*, and *Npy5r*
DBA/2J	Acute	+	Response to abiotic stimulus (0009628)	15	9.8 × e^-04^	*Cryab*, *Dnajb1*, and *Cdkn1a*
DBA/2J	Acute	+	Response to temperature stimulus (0009266)	5	1.7 × e^-03^	*Hspa8*, *Hspa1b*, and *Cryab*
C57BL/6J	Chronic	-	Cellular lipid metabolism (0044255)	17	1.95 × e^-04^	*Vldr*, *Sc5d*, and *Acsl3*
SWR/J	Acute	+	Glucose transport (0015758)	4	2.0 × e^-03^	*Stxbp3a*, *Stxbp4*, and *Aps*
SWR/J	Chronic	-	Cell communications (0007154)	25	2.3 × e^-03^	*Plcd4*, *Gjb1*, and *Utrn*
129P3/J	Acute	-	Neuron differentiation (0030182)	5	2.4 × e^-03^	*Olig2*, *Gli3*, and *Ablim1*
SWR/J	Acute	+	Carboxylic acid metabolism (0019752)	16	1.4 × e^-03^	*Cpt1a*, *Cpt2*, and *Crot*
C57BL/6J	Chronic	+	Negative regulation of biological process (0048519)	12	7.5 × e^-03^	*Zbtb16*, *Sgk3*, and *Adrb1*
SWR/J	Acute	-	Nervous system development (0007399)	7	9.9 × e^-03^	*Olig2*, *Rgma*, and *Ugt8a*
129P3/J	Chronic	+	Intracellular signaling cascade (0007242)	12	1.0 × e^-02^	*Ppm1*, *Dab1*, and *Snx24*

The complete results of the Gene Ontology (GO) analysis are presented in Additional data file 3. ^a^(+) increased mRNA abundance in response to morphine; (-) decreased mRNA level. ^b^Statistical significance of functional over-representation was computed using Fisher's exact test.

In the C57BL/6J mice, a single morphine injection induced the transcription of genes classified under several GO terms associated with apoptosis (GO: 0042981, GO: 0006915, GO: 0043066, GO: 0043066, GO: 0043068, and GO: 0006916), for example regulation of apoptosis (GO: 0042981; 11 transcripts; *P *= 0.00001). However, the neuronal functions of genes from this class, such as TSC22 domain family 3 (*Tsc22d3*/*Dsip1*/*Gilz*) or pleckstrin homology domain containing family F (*Plekhf1*), are not fully understood and might not be strictly associated with cell apoptosis. Acute morphine enhances the transcription of several functional groups of genes that are related to metabolism, including carboxylic acid metabolism (GO: 0019752) in DBA/2J (17 transcripts; *P *= 0.003) and SWR/J (16 transcripts; *P *= 0.001), as well as carbohydrate transport (GO: 0008643) in DBA/2J (five transcripts; *P *= 0.005). Proteins with transferase activity, including methionine adenosyltransferase II, α (*Mat2a*) and carnitine *O*-octanoyltransferase (*Crot*), were contained within these groups. Genes involved in glucose transport (GO: 0015758) were also upregulated in DBA/2J (four transcripts; *P *= 0.003), C57BL/6J (three transcripts; *P *= 0.005), and SWR/J (four transcripts; *P *= 0.002), including syntaxin binding proteins 3A (*Stxbp3a*) and 4 (*Stxbp4*). Moreover, morphine induced a group of transcripts involved in response to temperature stimulus (GO: 0009266). Over-representation of transcripts associated with response to temperature was observed after acute injection in DBA/2J (five transcripts; *P *= 0.002) and SWR/J (four transcripts; *P *= 0.009), but also after chronic treatment in DBA/2J (three transcripts; *P *= 0.005). For instance, gene transcription of heat shock proteins *Hspa1a *and *Hspa1b *was increased.

Acute injection of morphine downregulated genes that are involved in nervous system development (GO: 0007399) in 129P3/J (six transcripts; *P *= 0.006) and SWR/J (seven transcripts; *P *= 0.01) mice. However, the function of the majority of these genes, for example RGM domain family member A (*Rgma*) or myocyte enhancer factor 2C (*Mef2c*), in the adult brain is poorly characterized. In addition, a similar group of genes related to neuron differentiation (GO: 0030182) was downregulated in 129P3/J strain (five transcripts; *P *= 0.002).

Chronic morphine enhances gene expression of factors that participate in intracellular signaling cascades (GO: 0007242; 12 transcripts; *P *= 0.01) in 129P3/J mice, including disabled homolog 1 (*Dab1*) and protein phosphatase 1 (*Ppm1*) genes. Repeated morphine administration caused a reduction in mRNA abundance of genes that are involved in cellular lipid metabolism (GO: 0044255; 17 transcripts; *P *= 0.0002) in the C57BL/6J strain. Transcripts for very-low-density lipoprotein receptor (*Vldlr*) and sterol-C5-desaturase (*Sc5d*) were downregulated. Furthermore, mRNA levels of genes that are implicated in the transmission of nerve impulse (GO: 0019226) were found to be decreased after prolonged morphine treatment in three of four strains: C57BL/6J (ten transcripts; *P *= 0.0009), DBA/2J (five transcripts; *P *= 0.01), and 129P3/J (five transcripts; *P *= 0.009). Genes encoding synaptic receptors neuropeptide Y receptor Y5 (*Npy5r*), glutamate receptor ionotropic kainate 2 (*Grik2*), and GABAa receptor subunit (*Gabrg1*) were identified among this group.

**Figure 4:**
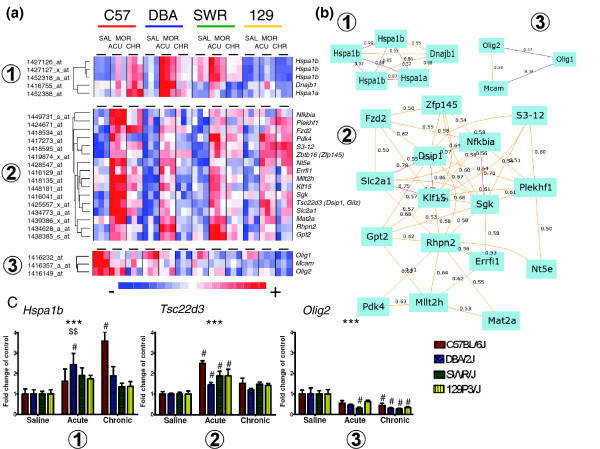
Morphine-induced co-regulation of gene transcription. **(a) **Clusters of genes co-expressed with (1) *Hspa1b*, (2) *Tsc22d3 *(*Dsip1*), and (3) *Olig2 *after morphine treatment (SAL, control group; MOR, acute [ACU] and chronic [CHR] morphine groups). Colored rectangles represent expression levels of the gene indicated by the probe set on the left and gene symbol on the right. Intensity of the color is proportional to the fold change as indicated on the bar below the cluster image. **(b) **Network graphs of transcripts co-regulated with (1) *Hspa1b*, (2) *Tsc22d3 *(*Dsip1*), and (3) *Olig2 *were generated using the gene-to-gene correlation tool in WebQTL (Hippocampus Consortium M430v2 Dec05 PDNN). Correlation coefficients for each pair of transcripts are indicated beside the line. The *Hspa1b *gene was represented by three different probe sets on the microarray. **(c) **Confirmation of morphine induced regulation of expression of three selected genes by quantitative real-time reverse transcription polymerase chain reaction (qPCR). Results are presented as fold change over control group with standard error (*n *= 6 to 9). Significant main effects from multivariate analysis of variance for morphine treatment (***P < 0.001) and interaction (^$$^*P *< 0.01) are indicated. Difference between morphine-treated and control groups was analyzed using Bonferroni *post hoc *test (^#^*P *< 0.05).

Several of the identified genes exhibited strong strain-specific changes in mRNA abundance. Differences in response were confirmed using the qPCR method. In strain DBA/2J, the level of heat shock protein 1B (*Hspa1b*) mRNA was increased after acute morphine by about 2.5-fold over control. Repeated treatment significantly reduced this effect in DBA/2J mice. On the contrary, the C57BL/6J strain exhibited a greater than threefold induction until after chronic treatment (Figure 4). Strain-dependent regulation of nuclear factor of kappa light chain gene enhancer in B-cells inhibitor α (*Nfkbia*) and dual specificity phosphatase 14 (*Dusp14*) transcripts was also verified by qPCR. Uniquely in the C57BL/6J strain, abundance of *Nfkbia *mRNA was increased almost twofold after acute morphine compared with control. *Dusp14 *expression was also upregulated only in C57BL/6J; a more than twofold increase was detected after chronic administration (Additional data file 4). Strain differences in transcriptional response were also observed for TSC22 domain family 3 (*Tsc22d3*/*Dsip1*/*Gilz*) and CCAAT/enhancer binding protein C/EBP delta (*Cebpd*). Both genes were strongly upregulated after acute morphine in C57BL/6J. However, mRNA level of *Tsc22d3 *was also noticeably elevated in the three other strains (Figure 4c).

### Genes co-regulated by morphine treatment

Three genes exhibiting substantial changes in expression after morphine treatment were selected as potential markers of biologic processes coordinated at the transcriptional level (*Hspa1b*, *Tsc22d3*, and *Olig2*). Prediction of co-expression of genes was verified using gene-to-gene correlation on the independent dataset. Analyses conducted in a large panel of BXD recombinant inbred (RI) strains (86) presented an opportunity to identify associative networks between transcripts [16]. Therefore, we conducted a search for genes with putative common mechanisms of transcriptional regulation in response to morphine using trait correlation analysis implemented in the WebQTL database.

Heat shock protein 70 (*Hspa1b*) was classified by GO analysis into functional groups associated with response to temperature and cellular stress. Thirty-four probe sets exhibited high positive correlations (*r *> 0.6, *n *= 86) with *Hspa1b *across the BXD RI panel, and four transcripts from this list were also significantly regulated by morphine. Expression of *Hspa1b *was highly correlated with mRNA levels of other heat shock proteins, namely heat shock protein 72 (*Hspa1a*) and heat shock protein 40 (*Dnajb1*). Inter-strain differences in response to acute and chronic morphine between C57BL/6J and DBA/2J were similar for all genes in this group. However, in the SWR/J mRNA profile, *Hspa1b *was noticeably different from *Hspa1a *and *Dnajb1*. Interestingly, no changes in expression of any of these genes were observed in the 129P3/J strain (Figure 4).

An association between TSC22 domain family 3 (*Tsc22d3*) and regulation of apoptosis was identified by GO analysis. Our further literature search yielded data that implicate the glucocorticoid receptor (GR) as a potential modulator of *Tsc22d3 *transcription [17]. Expression of the *Tsc22d3 *gene in the BXD RI panel exhibited a strong positive correlation with 146 transcripts. Unexpectedly, seven genes at the top of this list (*Sgk*, *Klf15*, *Fzd2*, *Gpt2*, *Rhpn2*, and *Nt5e*), characterized by a very high level of correlation (*r *> 0.72, *P *< 10^-16^, *n *= 86) also had a similar profile after morphine treatment. Moreover, from the list of 146 probe sets, 17 transcripts exhibited co-expression with *Tsc22d3 *in the BXD RI panel and appeared to be co-regulated after morphine treatment. In addition, this pattern in transcriptional response to morphine was most evident in the C57BL/6J strain (Figure 4).

Functional classification implicated oligodendrocyte-specific bHLH transcription factor 2 (*Olig2*) in nervous system development and neuron differentiation, in particular the development of motoneurons and oligodendrocytes. *Olig2 *expression in the BXD RI panel was positively correlated only with seven probe sets. This list includes one gene, melanoma cell adhesion molecule (*Mcam*), that is regulated by morphine with an analogous profile to *Olig2 *and decrease in mRNA level. In addition, *Olig1*, which had a similar regulation pattern after morphine treatment, was added manually to this group because functional connections with *Olig2 *are well described.

### Involvement of glucocorticoid receptor in transcriptional response to morphine

**Figure 5:**
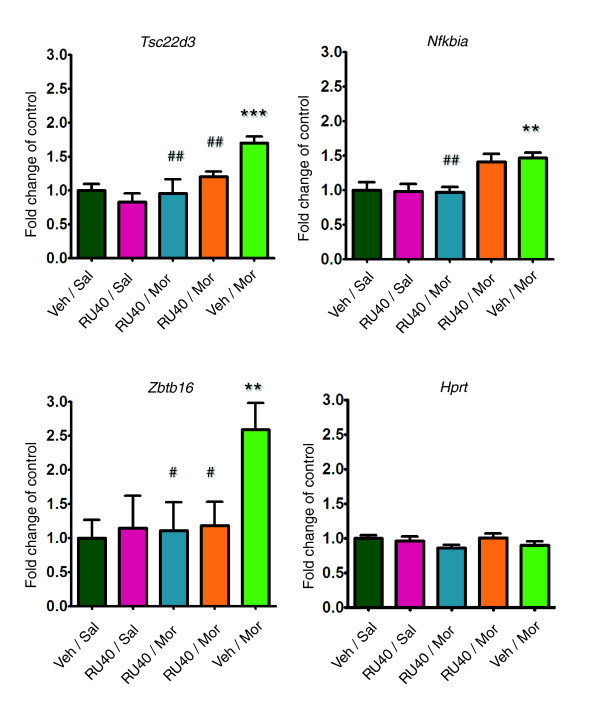
Involvement of glucocorticoid receptor in transcriptional response to morphine. Inhibition of morphine-induced transcription of selected genes by an antagonist of the glucocorticosteroid receptor, namely RU486. Five experimental groups were compared (see Materials and methods): vehicle and acute subcutaneous morphine 20 mg/kg (Veh/Mor); morphine preceded by injection of 20 mg/kg RU486 (RU20/Mor); morphine preceded by injection of 40 mg/kg RU486 (RU40/Mor); and control groups injected with saline and RU486 (RU40/Sal) or saline and vehicle (Veh/Sal). Gene expression in striatum of C57BL/6J mice was analyzed 4 hours after morphine treatment. The results of quantitative real-time reverse transcription polymerase chain reaction (qPCR) are presented as fold change of control group (Veh/Sal) with standard error. Differences between groups were analyzed by analysis of variance ANOVA following Bonferroni multiple comparison correction (*n *= 5 to 6; **P < 0.01, ***P < 0.001 versus control group; ^#^*P *< 0.05, ^##^*P *< 0.01 versus morphine).

The qPCR method was used to evaluate the influence of GR blockade on acute morphine-induced gene transcription of selected genes (*Tsc22d3*, *Nfkbia*, *Zbtb16*, *Sgk*, and *Fzd2*). All five genes exhibited marked co-regulation by morphine treatment and co-expression in the BXD RI panel (Figure 4). Changes in striatal gene expression were evaluated 4 hours after a single administration of morphine (20 mg/kg, subcutaneously). Morphine-induced increases in transcription of these genes were attenuated by administration of GR receptor antagonist RU486 30 min before morphine. Dose-dependent reductions in the increase in gene expression of *Tsc22d3 *and *Zbtb16 *were observed after administration of both tested doses of RU486 (20 and 40 mg/kg, intraperitoneally). In the case of *Nfkbia*, however, morphine-related induction of transcription was significantly attenuated only by the higher dose (Figure 5). Moreover, acute morphine induction of two other genes, namely *Sgk *and *Fzd2*, also decreased toward the control level after blockade of GR receptor (20 mg/kg RU486; data not shown). RU486 treatment did not alter basal mRNA levels of the selected genes as compared with those in the vehicle-treated control group. The results of the present study indicate that GR is involved in the expression of several morphine-responsive genes.

### Modulation of behavioral effects of morphine by glucocorticoid receptor blockade

**Figure 6:**
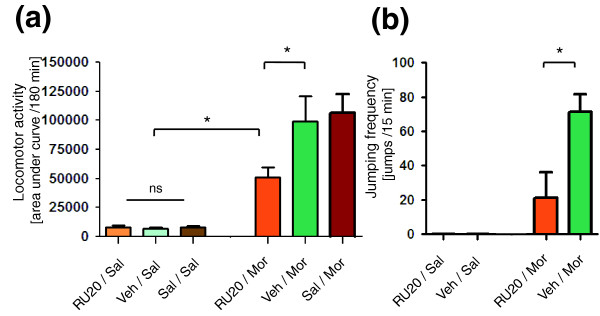
Modulation of behavioral response to morphine by RU486 pretreatment. **(a) **Effects of acute morphine administration (20 mg/kg, subcutaneously) on locomotor activity were analyzed in glucocorticoid antagonist-treated C57BL/6J mice. Mice were injected with 20 mg/kg RU486 (RU20). After 30 min, the mice were injected with morphine (Mor) or saline (Sal). Column bars correspond to the mean number of photocell counts per group with standard error (*n *= 6 to 8) collected from 0 to 180 min after administration of morphine. **(b) **Influence of glucocorticoid receptor antagonist pretreatment on the development of opioid physical dependence was analyzed in C57BL/6J mice. RU486 (20 mg/kg) was administered 30 min before acute injection of morphine (100 mg/kg, subcutaneously). The next day animals were injected with an additional dose of morphine (20 mg/kg, subcutaneously) following by the injection of naloxone (15 mg/kg, subcutaneously). The level of morphine dependence is presented as mean naloxone-precipitated withdrawal jumping response (number of jumps per 30 min after naloxone injection) with standard error (*n *= 5 to 6). Control animals were treated with vehicle (Veh) and saline (Sal). The data presented in panels a and b were analyzed using analysis of variance following Tukey's *post hoc *test (*P < 0.05; ns, not significant).

The influence of GR blockade on locomotor stimulant effects of morphine and development of physical dependence was evaluated in the C57BL/6J strain. Acute morphine (20 mg/kg, subcutaneously) treatment induced typical hyper-locomotion (Figure 6a). Effects of morphine in the GR antagonist RU486-treated (20 mg/kg) mice were significantly attenuated compared with those in the vehicle-treated animals (*P *< 0.05). Control groups of animals (treated only with RU486, saline, or vehicle) did not exhibit locomotor stimulation during the experiment; there were also no statistically significant differences between these groups. RU486 treatment did not alter basal locomotor activity (Figure 6a).

A single dose of RU486 (20 mg/kg) was administered 30 min before an injection of morphine (100 mg/kg, subcutaneously). On the next day animals were injected with an additional dose of morphine (20 mg/kg, subcutaneously) followed by injection of naloxone (after 3 hours; 15 mg/kg). This treatment scheme resulted in robust physical dependence in morphine-treated mice, as revealed by the mean number of jumps (71.5 ± 9.9 jumps per 15 min). RU486 pretreatment significantly suppressed naloxone-precipitated withdrawal jumping response (21 ± 15.1 jumps per 15 min) compared with the morphine-treated group (*P *< 0.05; Figure 6b). Other signs of opioid withdrawal such as defecation and urination were also attenuated in RU486-treated mice.

## Discussion

Comparison of striatal gene expression profiles of the selected strains of mice indicated that expression of 2,870 transcripts was affected by genetic background. The majority of the detected differences in the mRNA levels were highly statistically significant but relatively small in the magnitude. A large degree of disparity between inbred mouse strains in the brain transcriptome is in accordance with previously published data [18]. Furthermore, complex strain-specific and region-specific expression patterns of a number of transcripts were recently reported [19]. Consequently, differences in mRNA distribution restricted to subregions or specific cellular populations in striatum are among the possible explanations for relatively small fold differences. On the other hand, our earlier studies suggested the presence of inter-strain differences in the level of mRNA variants for the number of genes (for example, *Atp1a2 *and *Comt*) [14]. The results obtained here extend our previous observations and present a broader list of potential candidate genes for further study. The influence of differences in particular genes on the genome-phenome interaction remains to be elucidated.

In the present study, we analyzed the effects of acute and chronic morphine treatment. Genotype-independent influences of morphine treatment on gene expression profile in the striatum were evaluated in all four mouse strains. The degree of alteration in gene expression (618 transcripts) was significantly less than the above-mentioned differences between the strains in terms of basal transcript levels.

Acute morphine injection increased the mRNA level of a large group of genes, including the functional groups of transcripts associated with response to abiotic and temperature stimuli. These transcriptional responses are potentially related to common physiologic effects induced by opioids in mice [10]. Both acute and chronic morphine administration resulted in mRNA upregulation of genes classified using GO terms as factors involved in the regulation of cellular processes and apoptosis, whereas mRNA abundance of genes linked to nervous system development and cell-cell communication decreased. However, we consider GO analysis as providing an indication of functional association between groups of genes, rather demonstrating a direct connection with each process described by a GO term. For instance, serum/glucocorticoid-regulated kinase 3 (*Sgk3*) was classified as a gene associated with apoptosis, whereas the expression of *Sgk3 *in brain was also associated with memory consolidation [20]. Our data include changes in the expression of genes with functional importance to the prolonged effects of morphine, such as plasminogen activator (*Plat*) and FK506-binding protein 5 (*Fkbp5*). Morphine upregulation of *Plat *mRNA was linked to drug-related reward properties [21], whereas the inhibition of *Fkbp5 *was found to prevent symptoms of opioid withdrawal syndrome [22]. In our experiments, morphine-induced changes in striatal gene expression of components of intracellular signaling pathways, for example calcium/calmodulin-dependent protein kinase I γ (*Camk1g*) and the receptor for Wnt signaling proteins frizzled homolog 2 (*Fzd2*), were identified. However, the previously described downregulation of mRNAs for cytoskeleton-related genes and genes involved in mitochondrial respiration after acute morphine was not confirmed [23]. These discrepancies may result from the experiments being conducted at different time points.

In comparison with acute morphine, repeated administration caused a large decrease in mRNA levels of many genes, including the over-represented group of histone transcripts involved in nucleosome assembly and chromatin remodeling. However, remodeling of chromatin was identified as important regulatory mechanism of cocaine-induced plasticity in striatum [24]. Moreover, one of the genes with decreased mRNA level after repeated morphine treatment, namely oligodendrocyte transcription factor 2 (*Olig2*), was identified as an essential transcriptional regulator in neuron and oligodendrocyte specification [25] and was recently postulated to be involved in susceptibility to schizophrenia [26]. In summary, these observations may suggest that chronic morphine-induced alterations in the striatum are related to cell plasticity and structural changes.

One of the main aims of the present study was to investigate genotype-dependent differences in transcriptional response induced by morphine. Comparison of the number of genes regulated by morphine across the four strains has indicated that acute response is markedly greater in DBA/2J and SWR/J mice than in C57BL/6J and 129P3/J mice. DBA/2J and SWR/J strains exhibited over-representation of metabolism-related genes across the list of regulated transcripts. Both strains exhibited greater hypothermic response to single morphine administration [10]. Furthermore, acute morphine induces increase in locomotor activation in C57BL/6J and 129P3/J mice, but not in DBA/2J and SWR/J [10]. The findings suggest that strain differences in over-expression of metabolism-related genes may be associated with behavioral responses to morphine. These changes in gene expression might be among the possible factors that determine development of morphine-related traits. For instance, DBA/2J and SWR/J strains exhibited low morphine preference, measured as oral self-administration of morphine [12].

The obtained profiles of gene expression indicate that some of the alterations in transcription appear to be related to the physiologic state of the whole body. Co-expression of three genes that are typically associated with cellular stress and temperature stimulus (*Hspa1a*, *Haspa1b*, and *Dnajb1*) was detected after morphine treatment. Further analysis of heat shock protein 1b gene expression revealed inter-strain differences in response. The greatest induction of heat shock protein (HSP) mRNAs after acute morphine administration was observed in DBA/2J, whereas after chronic morphine treatment it was observed in C57BL/6J mice. As mentioned above, acute morphine produces severe hypothermia in mice [10]. However, the inter-strain differences in profile of HSP mRNA expression after acute morphine were negatively related to changes in body temperature. On the other hand, opioid-induced hypoxia caused by respiratory depression with different escalation in the strains might be involved in expression of HSP genes. Increased expression of Hsp70 mRNA in rat brain was suggested to be a protective mechanism against the harmful effects of opiates [27]. Interestingly, different responses in gene expression of Hsp70 were observed between rats after active and passive morphine administration [28]. The presented results support previous data and implicate regulation of HSP gene expression as a potential marker of physiologic alterations of homeostatic processes induced by morphine in brain.

**Figure 7:**
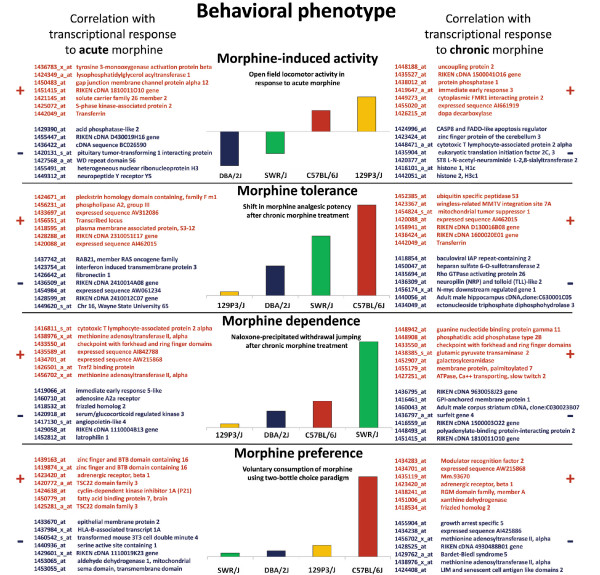
Morphine-induced changes in gene expression correlated with the behavioral response. Transcriptional and behavioral responses to morphine were compared across the four inbred strains of mice with specific opioid-related phenotype (C57BL/6J, DBA/2J, 129P3/J, and SWR/J). Behavioral data were gathered as described in the Materials and methods section. The results were obtained by using Pearson correlation (Additional data file 5). Correlation between each opioid-related trait and transcriptional response to acute morphine is presented on the left panel along with chronic morphine on the right panel. Positive correlations between changes in gene expression and behavioral response are highlighted by red color (+), and negative ones are highlighted by the blue color (-).

To identify transcriptional response associated with specific opioid-related traits, changes in mRNA level were correlated with morphine-induced behavioral traits (Additional data file 5). Severe symptoms of opioid withdrawal observed in SWR/J mice were associated with strong transcriptional activation of cytotoxic T lymphocyte-associated protein 2 (*Ctla2a*) and methionine adenosyltransferase II (*Mat2a*) genes after acute administration of morphine, whereas changes in gene expression of adenosine A2a receptor (*Adora2a*) were negatively related to the level of morphine physical dependence across the four inbred mouse strains (Figure 7). A role of functional activation of the adenosine A2a receptor in opioid withdrawal was previously reported [29].

Decreased expression of several genes linked to the transmission of nerve impulse after chronic treatment may be connected with the development of tolerance to effects of opioids observed in C57BL/6J strain, including morphine-responsive genes encoding NPY receptor (*Npy5r*) and GABAa receptor subunit (*Gabrg1*). A moderate transcriptional response to acute treatment and a large decrease in the mRNA abundance of many genes after chronic morphine in C57BL/6J occur together with an enhanced preference [30] for and intake of morphine in this strain [12]. Increased mRNA abundance of TSC22 domain family 3 (*Tsc22d3*) and zinc finger and BTB domain containing 16 (*Zbtb16*) transcripts was found to be positively related to morphine preference (Figure 7).

Strain differences in response to acute morphine were also detected in the level of nuclear factor of kappa light chain gene enhancer in B-cells inhibitor α (*Nfkbia*) mRNA. Significant induction of *Nfkbia *gene expression after 4 hours was detected in C57BL/6J mice. A similar profile of gene transcription was observed for *Tsc22d3*. It is well established that gene transcription of *Nfkbia *as well as *Tsc22d3 *can be mediated by GR and controlled by the level of glucocorticoids [17,31]. Nuclear receptor GR is a ligand-activated transcription factor that directly modulates transcription of downstream genes [32]. Therefore, it has been suggested that glucocorticoids are potentially factors that are responsible for the detected increase in expression of these genes. Furthermore, we have identified a relatively large group of genes with parallel expression profiles to that of *Tsc22d3 *(*Sgk*, *Klf15*, *Fzd2*, *Gpt2*, *Rhpn2*, *Nt5e*, and *Nfkbia*), which may indicate morphine-induced co-regulation of these genes.

Therefore, the putative involvement of glucocorticoids in the modulation of transcription of these genes was further studied using the GR antagonist RU486. Prior administration of RU486 blocked morphine-produced induction of *Tsc22d3*, *Zbtb16*, and *Nfkbia *mRNAs. The functional consequences of this transcriptional process remain unknown. However, it was previously shown that GR-dependent transmission influences the behavioral response to morphine in rats [33,34]. The present study demonstrated that GR blockade can inhibit an increase in locomotor activity following morphine administration in C57BL/6J mice. Influence of the GR antagonist on the relatively fast stimulatory effect of morphine on locomotion may suggest the involvement of a nongenomic mechanism. Nevertheless, several lines of evidence indicate the GRs also play an important role in the development of opioid-related phenotype. Secretion of glucocorticoids is involved in sensitization to activatory effects of morphine [35]. On the other hand, substantial attenuation of the morphine analgesic tolerance after administration of RU486 has been reported in rats [36]. Furthermore, our findings indicated involvement of GRs in the development of morphine physical dependence. Thus, the GR antagonist appears to modify both the behavioral and transcriptional responses to morphine. Therefore, GR-mediated gene expression in the striatum may play a role in the chronic effects of morphine and could be involved in the formation of drug-associated behavior. This hypothesis is also supported by the recent findings that GRs in the nucleus accumbens are necessary for the development of conditional place preference for morphine [37]. The increase in glucocorticoid levels induced by acute stress facilitates the consolidation of memories associated with emotional stimuli [38]. The greatest induction of the genes putatively regulated by glucocorticoids was detected in C57BL/6J mice, an inbred strain characterized by high preference for morphine as well as alcohol [12,39].

It is likely that induction of glucocorticoids and further GR-dependent gene expression might also enhance learning of drug-related stimuli. It was suggested that serum-glucocorticoid-inducible kinase (*Sgk*) is involved in memory consolidation of hippocampus-dependent learning [40]. Also, implication of the *Nfkbia *regulatory protein of nuclear factor-κB in memory formation in mice has already been proposed [41]. Withdrawal from chronic morphine treatment induced changes in the mRNA abundance of *Sgk *and *Nfkbia *in rat prefrontal cortex [42] and mouse locus coeruleus [22]. Furthermore, alterations in expression of *Sgk *and *Nfkbia *were also observed after acute administration of ethanol [43-45]. However, shared transcriptional regulation of the genes mentioned here was not emphasized or directly associated with the action of glucocorticoids. The potential therapeutic use of GR antagonists in treatment for drug abuse was previously suggested [46]. The present study identified a group of GR-dependent genes that are regulated in response to morphine. Moreover, our results provide new insights into the morphine-induced mechanism of action of glucocorticoids in the brain.

## Conclusion

Comparison of morphine-induced changes in striatal gene expression across the four inbred mouse strains indicated new biologic mechanisms that are potentially involved in the action of morphine. The results describe strain differences in the magnitude of transcriptional response to acute treatment and in the degree of tolerance in gene expression observed after chronic morphine administration. Further profiling of gene expression and transcriptional activity are required to characterize fully the mechanisms of transcriptional regulation and the dynamics of changes in mRNA abundance induced by opioids in mice. As a final point, the obtained results indicated the participation of several novel molecular factors in the effects of morphine and suggested a transcriptional basis for the well known association between glucocorticoids and opioid addiction.

## Materials and methods

### Mice

Adult male (8 to 10 weeks old) 129P3/J (000690), DBA/2J (000671), C57BL/6J (000664), and SWR/J (000689) mice (Jackson Laboratory, Bar Harbor, ME, USA) were housed six per cage, under a 12 hour dark/light cycle, with free access to food and water. Animals weighing 20 to 30 g were used throughout the experiments. The animal protocols used in the study were approved by the local Bioethics Commission at the Institute of Pharmacology, Polish Academy of Sciences (Krakow, Poland).

### Morphine treatment

Morphine (morphine hydrochloride; Polfa, Kutno, Poland) was administered subcutaneously. Experimental groups (control, and acute and chronic morphine) consisted of nine animals from each strain. To obtain the most reliable comparison, control and acute morphine groups received injections of saline for 4 days at the same time schedule as the chronic group received morphine (Figure 1). On day 5, acutely treated animals were injected with a single dose of morphine and killed by decapitation after 4 hours. Chronically treated animals were injected with increasing doses of morphine for 5 days. Mice received morphine thrice daily (09:00 hours, 13:00 hours, and 17:00 hours) for 4 days using a dosing schedule of 10, 20, 40, and 40 mg/kg morphine on days 1, 2, 3, and 4, respectively. On the last day, a final morphine dose of 40 mg/kg was administered, and 4 hours after the last injection the animals were killed. Mice in control groups were killed 4 hours after the last injection of saline. The dose scheme and time schedule were used in order to maximize strain differences in response to morphine [10,13,47].

### RU486 treatment

Five groups of six C57BL/6J mice were used in the gene expression experiment with morphine and RU486 (Sigma-Aldrich, St. Louis, MO, USA) treatment. The control group was injected subcutaneously with saline and intraperitoneally with 20% (2-Hydroxypryl)-β-cyclodextrine (vehicle for RU486; Sigma). RU486 (20 or 40 mg/kg in vehicle, intraperitoneally) was administered 30 min before morphine administration (20 mg/kg, subcutaneously). The doses of RU486 were selected based on a previous report that showed the effects of peripheral administration on the central nervous system [48]. For the qPCR experiment, animals were killed 4 hours after the morphine or saline injection.

### Behavioral testing

An independent pool of C57BL/6J mice was used in the behavioral experiments (five to eight animals per experimental group). In the locomotor activity test mice were individually placed in the center of a test cage containing photocells (20 cm × 10 cm × 12 cm). The photocells recorded the number of beam interruptions in the horizontal plane every 15 min over a 4.5 hour period. After 1 hour, the mice were injected with RU486 (20 mg/kg in vehicle, subcutaneously). The control group was injected with saline and vehicle. The influence of RU486 (20 mg/kg in vehicle, subcutaneously) on basal locomotor activity was also evaluated. After 30 min, mice were injected with saline or morphine (20 mg/kg, subcutaneously). Measurement of locomotor activity was performed for the next 3 hours. The results were calculated as an area under curve.

Physical dependence was induced by acute injections of morphine (100 mg/kg, subcutaneously). A single RU486 dose (20 mg/kg in vehicle, subcutaneously) was administered 30 min before the injection of morphine. On the second day mice received an additional subcutaneous dose of 20 mg/kg morphine, followed by a single naloxone dose (15 mg/kg, subcutaneously) 3 hours later. Mice did not receive RU486 on the day of the test. For mice in control groups, saline was substituted for morphine. Measurement of naloxone-precipitated withdrawal was performed as described in previous studies [13]. Mean jump frequency per 15 min was used as the measure of dependence. Behavioral data was analyzed by two-way analysis of variance (ANOVA) followed by Bonferroni *post hoc *test, with RU486 treatment and morphine treatment as the main factors.

### Tissue collection and RNA isolation

After decapitation, brains were removed from the skulls and dissected rapidly. Samples containing the rostral part of caudate/putamen plus the nucleus accumbens (referred to as the striatum) were collected. The samples were placed in individual tubes with the tissue storage reagent RNA *later *(Qiagen Inc., Valencia, CA, USA), frozen on dry ice, and stored at -70°C until RNA isolation. Samples were thawed at room temperature and homogenized in 1 ml Trizol reagent (Invitrogen, Carlsbad, CA, USA). RNA isolation was performed in accordance with the manufacturer's protocol. Quality of the total RNA was assessed by the intensity of 28S and 18S bands after denaturating agarose electrophoresis with SybrGold staining (Molecular Probes, Inc., Eugene, OR, USA) and by the spectrophotometric ratio A260/A280 (1.9 to 2.1). RNA concentration was measured using the fluorescent reagent RiboGreen (Molecular Probes, Inc.).

### Microarray hybridization

Total RNA from three animals was pooled and further purified using the RNeasy Mini Kit (Qiagen Inc.). The quality of RNA was additionally determined by chip-based capillary electrophoresis using RNA 6000 Nano LabChip Kit and Agilent Bioanalyzer 2100 (Agilent, Palo Alto, CA, USA), and there was little evidence of degradation products in any of the total RNA samples. For each array, independent pools of RNA from three animals were prepared. Preparation of cRNA was performed according to the protocol provided by Affymetrix (Santa Clara, CA, USA). Total RNA (5 μg) derived from each pool was converted to double-stranded cDNA using the SuperScript System (Invitrogen) and an oligo(dT_24_) primer containing a T7 RNA polymerase promoter site (Genset Oligos, La Jolla, CA, USA). Biotin-labeled cRNA was synthesized from cDNA using a BioArray High Yield RNA Transcript labelling Kit (ENZO, Diagnostics, Farmingdale, NY, USA) and purified using a GeneChip Cleanup Sample Module (Qiagen Inc.). The yield of the *in vitro *transcription reaction was determined by product absorbance at 260 nm measured using NanoDrop ND-1000 (NanoDrop Technologies, Inc., Montchanin, DE, USA), and the size of cRNA probes was evaluated by using the RNA 6000 Nano LabChip Kit (Agilent).

Following labeling, samples were hybridized to the GeneChip Test3 array (Affymetrix) for quality control. Fragmented cRNA (15 μg) was used for hybridization to the GeneChip Mouse Genome 430 2.0 arrays (Affymetrix). Arrays were washed and stained with streptavidin-phycoerythrin (Merck, Darmstadt, Germany) in Fluidic Station 400 (Affymetrix), in accordance with the standard protocol of the manufacturer. The arrays were scanned using the GeneChip Scanner 3000 (Affymetrix). Three biologic replicates of the microarrays were prepared per experimental group of animals for a total of 36 arrays.

### Microarray quality control and normalization

The expression data were processed using the GeneChip Operating Software (Affymetrix) to generate MAS5 CEL files. Chip quality was assessed using R 2.3.0 with the simpleaffy package [49,50]. Quality control data for all 36 microarray runs in the experiment were obtained using the MAS5 algorithm. The mean 3'/5' degradation ratio for control probe sets of housekeeping genes was measured: *Gapdh *(AFFX-GapdhMur/M32599) -0.2 (-0.39> ... <0.57) and *Actb *(AFFX-b-ActinMur/M12481) 0.57 (0.33> ... <1.24). Array normalization resulted in a mean scaling factor of 0.87 (± 0.57). The mean percentage of the present call was 51.34 (47.3> ... <54.63), and the mean intensity of the average background ranged from 27.9 to 76.9. Arrays determined to be acceptable were further analyzed to identify genes with altered expression patterns. Data was normalized using three different methods using RMAExpress 0.4.1 (RMA), dChip 2006 (MBEI), and PerfectMatch 2.3.3 (PDNN) software [51-53]. Quantile normalization was performed using RMA, PDNN, and MBEI algorithms. A model-based expression index was calculated using perfect match/mismatch (PM/MM) method. Multiple analysis strategies were used to identify the most robust changes in gene expression.

### Gene filtering and ranking

To remove genes that are regarded as not expressed in the analyzed brain tissue, probe sets with hybridization signals close to the background level were filtered out. The following criteria were applied for probe set detection: present call at least in 25% of PM/MM pairs and signal intensity greater than 6.64 (log_2 _data) in at least 25% of arrays, both measured using the MBEI algorithm. Statistical analysis was performed on the list of detected probe sets. Significance levels (*P *values) of differences between the groups were calculated for each probe set using a MANOVA for each of three pre-processing methods. Correction for multiple testing was applied separately for each of the main factors from MANOVA by controlling percentage FDR. The FDR was computed using the p.adjust function in R software [54]. Gene ranking was based on FDR levels of three selected normalization methods. A probe set scored 1, 2, or 3 points if it achieved cut-off at *P *< 0.01, *P *< 0.001, or *P *< 0.0001, respectively, for each of the three methods. Two criteria for threshold point were used: the first produced a list of probe sets with rank greater than 3 (*P *< 0.01 in all the three normalization methods) and the second list with rank greater than 7 (*P *< 0.0001 in two of the methods and at least 0.001 in the third). An applied approach that takes into account the level of agreement between the three methods of normalization was previously established [14].

To assess the contribution of strain and morphine treatment effects, we performed multiple regression analysis of expression values, with strain and treatment as main effect predictors using the lm function in R. Contributions of the effects were determined for each gene separately.

Hierarchical clustering was performed with dChip software using Euclidean distance and average linkage method. Relative expression levels and fold change measures were computed from MBEI PM/MM data. To simplify the description of microarray data, results from each probe set were assumed to be the level of mRNA abundance of the transcript. Probe sets on the Affymetrix microarray were designed to detect specific transcripts and are adequately annotated. However, it must be noted that for some probe sets this assumption may not be fulfilled. Therefore, every list of probe sets obtained in the analyses is included in Additional data files. Direct probe mapping against publicly available mRNAs/cDNA sequences was done by using mouse BLAT tool for the UCSC genome browser [55].

### Gene Ontology analysis

The functional annotation analysis tool DAVID 2006 was used to identify over-represented ontologic groups among the gene expression profiles and to group genes into functional classifications [56]. The list of 21,467 detected probe sets was uploaded as a background list. Over-represented GO terms (GOTERM_ALL level) were defined as having at least three transcripts and *P *= 0.01, under Fisher's exact test.

### Correlation between genomic and behavioral response to morphine

Relationships between the transcriptional response to morphine and opioid-related traits were studied using correlation analysis. Gene expression results were compared with the previously published behavioral data for the four inbred mouse strains. The first line of evidence demonstrated open field locomotor activity in response to acute morphine (16 mg/kg), measured from 0 to 30 min after injection, and compared with a saline control group (C57BL/6J = 4,116, 129P3/J = 5,880, SWR/J = -1,500, and DBA/2J = -3,088); this was reported by Belknap and coworkers [10]. Second, morphine tolerance has been demonstrated, calculated as a shift in morphine analgesic potency after chronic treatment (C57BL/6J = 7.2, SWR/J = 5, DBA/2J = 2.9, and 129P3/J = 0.8); this was reported by Kest and colleagues [47]. Third, physical dependence on morphine has been demonstrated following chronic treatment, as indicated by a naloxone precipitated withdrawal jumping response (SWR/J = 204 jumps/15 min, C57BL/6J = 68 jumps/15 min, DBA/2J = 45 jumps/15 min, and 129P3/J = 7 jumps/15 min); these data were reported by Kest and coworkers [13]. The fourth line of evidence demonstrated voluntary morphine consumption in two-bottle choice paradigm (C57BL/6J = 134 mg/kg per day, 129P3/J = 24 mg/kg per day, DBA/2J = 15 mg/kg per day, and SWR/J = 6 mg/kg per day); these ata were reported by Belknap and colleagues 1993 [12]. The expression level of genes altered by acute and/or chronic morphine (699 probe sets, obtained by MBEI PM/MM) and behavioral data were normalized using z-score transformation. Associations were computed using the Pearson's correlation. Input data, annotations for probe sets, and the obtained results are included in Additional data file 5.

### Identification of genes co-expressed in response to morphine

Genes with related patterns of expression were selected from the list of probe sets with significant changes after morphine treatment. To confirm associations between the transcripts, additional analysis was performed on an independent set of data. The WebQTL database was used to assemble transcripts into groups with potentially common regulatory mechanisms [16]. A hippocampus gene expression dataset with results from 86 BXD RI strains (Hippocampus Consortium M430v2 Dec05 PDNN) was applied as an easily accessible tool with relatively high ability to detect associations between genes. Selected probe sets for genes regulated by morphine were analyzed using the trait correlation WebQTL tool. Correlations were computed using Pearson's product-moment. The most significant top 200 results were obtained. Only probe sets with positive correlations (*r *> 0.6) were extracted and used in further analyses. A list of probe sets with significant changes after morphine and positive correlation across the BXD RI panel was generated. Network graphs were obtained with WebQTL interface using Pearson's product-moment and default parameters.

### Real-time PCR

Reverse transcription was performed using Omniscript reverse transcriptase (Qiagen Inc.) at 37°C for 60 min. qPCR reactions were performed using Assay-On-Demand Taqman probes (Additional data file 4), in accordance with the manufacturer's protocol (Applied Biosystems) and run on the iCycler device (BioRad) with the 3.0a software version. RT reactions were carried out in the presence of RNase inhibitor (rRNAsin; Promega, Madison, WI, USA) and oligo(dT_16_) primer (Qiagen Inc.). cDNAs were diluted 1:6 with H_2_O and for each reaction about 50 ng of cDNA synthesized from total RNA template from individual animals was used. To reduce the contribution of contaminating genomic DNA, primers were designed to span exon junctions. In addition, for each assay, control reactions without RT enzyme were performed. Amplification efficiency for each assay was determined by running a standard dilution curve. Expression of hypoxanthine guanine phosphoribosyl transferase 1 (*Hprt1*) transcript with a stable level between the strains and after the treatment was quantified to control for variation in cDNA amounts. The cycle threshold values were calculated automatically by iCycler IQ 3.0a software with default parameters. Abundance of RNA was calculated as 2^-(thresholdcycle)^. Data were analyzed by two-way ANOVA followed by Bonferroni *post hoc *test.

## Additional data files

The following additional data are available with the online version of this manuscript. Additional data file 1 is a table listing the results of two-way ANOVA (FDR < 1%). Additional data file 2 contains lists of probe sets of genes with expression altered by acute and chronic morphine (ANOVA; FDR < 1%). Additional data file 3 is a table listing the results of the GO analysis. Additional data file 4 contains the results of validation of microarray data obtained using the qPCR method. Additional data file 5 lists the complete results of correlation analysis between the transcriptional response to morphine and opioid-related traits (see Materials and methods).

## Supplementary Material

Additional data file 1 Lists of probe sets and gene names altered by strain (3457) and morphine treatment (661) as well as with significant interaction (48) are available as separate sheets. List of transcripts with differences in both the factors (178) was also included. In addition, genes reported in previous gene expression studies on morphine action in a brain are indicated. Click here for file

Additional data file 2 The results were obtained using fold change (>1.2) of gene expression level compared with the saline control group. Effects of morphine treatment were analyzed in all of the strains as well as separately in each of them. Numbers of probe sets correspond to those in Table 2. Click here for file

Additional data file 3 Presented are significant functional categories (GO terms) enriched with genes regulated in response to acute and/or chronic morphine, along with lists of probe sets and gene names classified to each GO category. Click here for file

Additional data file 4 Results for selected genes are presented as mean (± standard error) compared with the saline control group. List of Taqman assays used in qPCR experiments with ID and exon boundaries is included. Click here for file

Additional data file 5 The expression level (MBEI PM/MM algorithm) of genes altered by acute and/or chronic morphine (699 probe sets) and behavioral data were normalized using z-score transformation. Associations were computed using the Pearson's correlation. Click here for file
